# Flow Cytometry to Assess the Counts and Physiological State of *Cronobacter sakazakii* Cells after Heat Exposure

**DOI:** 10.3390/foods8120688

**Published:** 2019-12-16

**Authors:** Paloma Cal-Sabater, Irma Caro, María J. Castro, María J. Cao, Javier Mateo, Emiliano J. Quinto

**Affiliations:** 1 Department of Nutrition and Food Science, Faculty of Medicine, University of Valladolid, 47005 Valladolid, Spain; calsabater89@gmail.com (P.C.-S.); irma.caro@uva.es (I.C.); 2 Department of Nursery, Faculty of Nursery, University of Valladolid, 47005 Valladolid, Spain; mjcasalija@gmail.com (M.J.C.); mjcao@enf.uva.es (M.J.C.); 3 Department of Hygiene and Food Technology, Faculty of Veterinary Medicine, University of León, 24071 León, Spain; jmato@unileon.es

**Keywords:** flow cytometry, *Cronobacter sakazakii*, infant formulae, heat stress, compromised cells, thermal resistance

## Abstract

*Cronobacter sakazakii* is an opportunistic pathogen that is associated with outbreaks of neonatal necrotizing enterocolitis, septicaemia, and meningitis. Reconstituted powdered infant formulae is the most common vehicle of infection. The aim of the present study is to gain insight into the physiological states of *C. sakazakii* cells using flow cytometry to detect the compromised cells, which are viable but non-culturable using plate-based methods, and to evaluate the impact of milk heat treatments on those populations. Dead-cell suspensions as well as heat-treated and non-heat-treated cell suspensions were used. After 60 or 65 °C treatments, the number of compromised cells increased as a result of cells with compromised membranes shifting from the heat-treated suspension. These temperatures were not effective at killing all bacteria but were effective at compromising their membranes. Thus, mild heat treatments are not enough to guarantee the safety of powered infant formulae. Flow cytometry was capable of detecting *C. sakazakii*’s compromised cells that cannot be detected with classical plate count methods; thus, it could be used as a screening test to decrease the risk derived from the presence of pathogenic viable but non-culturable cells in this food that is intended for newborns’ nutrition.

## 1. Introduction

During the last decade, scientific interest has turned to *Cronobacter sakazakii*, formerly *Enterobacter sakazakii*, as a human pathogen. *C. sakazakii* is an opportunistic pathogen that is associated with outbreaks of neonatal necrotizing enterocolitis, septicemia, and meningitis [1,2,3]. The first virulence factors identified in *C. sakazakii* were enterotoxins [4]. Raghav and Aggarwal [5] identified a 66kDa toxin which was most active at pH 6, with the ability to be stable at 90 °C for 30 min, and potent cell toxicity (LD_50_ = 56 pg). This organism has a high case fatality rate in vulnerable infants in neonatal intensive care units and in surviving patients, severe neurological sequelae have occurred including hydrocephalus, quadriplegia, and developmental delay [2,6].

The International Commission for Microbiological Specifications for Foods [7] ranked *Cronobacter* spp. as a “severe hazard for restricted populations causing life-threatening or substantial chronic sequelae of long duration.” The FAO/WHO [8] noted that infants are the group at particular risk although *C. sakazakii* can cause invasive infection in all age groups. Reconstituted powdered infant formulae (PIF) and powdered milk are the most common vehicles implicated in neonatal *C. sakazakii* infections [3,9,10,11,12].

PIF could be easily contaminated because it is a non-sterilized product [3]. In hospitals, environmental contamination and temperature abuse of the reconstituted formula have been contributory factors [9,13]. The FAO/WHO [14] recommends cooling the temperature of boiled water not below 70°C for safe preparation of PIF. Heat stress can damage the bacterial cell wall and cause breakage of genomic DNA along with misfolding of cytoplasmic proteins [15]. However, bacteria can survive to sublethal heat stress and can also be adaptive by inducing a response to high environmental temperatures [16], which has been demonstrated for *Cronobacter* spp. [17].

The use of plate count methods to assess the number of stressed bacteria does not provide direct information on their physiological status [15,18]. This approach only detects cells that are able to form colonies but cannot detect metabolically active cells that do not form them [18,19], i.e., compromised—damaged or injured—bacteria [15,20,21,22]. In contrast, flow cytometry (FC) provides information on the physiological heterogeneity of bacterial populations [22,23,24,25] using fluorescence markers and direct optical devices to quantify the properties of single cells [26,27] and provides a fundamental advantage over conventional methods [28,29]. Compromised bacteria have special growth requirements due to their physiological state and include two types of cells, i.e., those that could be culturable through proper culture conditions and cells that are viable but non-culturable. To detect the former, it is necessary to adapt the culture conditions such as media, temperature, and time [30]. The most common strategy is to include a recovery step using a non-selective medium prior to the inoculation of the bacteria into the selective media. Some authors propose to use a plate count technique with both non-selective and selective culture media. Indeed, the difference between both counts would reflect the count of cells with sublethal damage [31,32]. Although non-selective methods are used, compromised viable but non-culturable cells cannot be detected by using classical plate count methods [33,34,35] and, unfortunately, from a public health point of view, they can retain their pathogenic potential.

Bacteria culture techniques are time-consuming and do not show the physiological state of the cells [36,37]. Therefore, the aim of the present study is to gain insight into the physiological states of *Cronobacter sakazakii* cells using flow cytometry in order to detect viable but non-culturable compromised cells and to evaluate the impact of mild heat treatments on those populations.

## 2. Materials and Methods

### 2.1. Culture Preparation

*Cronobacter sakazakii* ATCC 29544 was used [38,39,40,41]. In triplicate (in three different days) the strain was cultured twice in sterile Trypticase Soy Broth (TSB; Difco, BD Diagnostics, Spark, MD, USA) at 37 °C for 24 h to reach the stationary phase, with a concentration of ca. 10^9^ CFU/mL. The populations were counted after spreading 10 µL aliquots from serial dilutions onto TSA plates following the drop method [42] and were incubated at 37 °C for 24 h. A population of 1.1 × 10^9^ (±0.1 × 10^9^) CFU/mL was counted.

### 2.2. Dead-Cells Suspensions (DCS)

Dead-cells single-colour suspensions were prepared as controls for setting up the FC procedure following the manufacturer’s instructions of the staining kit that is used later (see below). Portions of 1 mL from the microorganism’s cultures were dispensed into microcentrifuge tubes, centrifuged at 10,000 *g* for 3 min to pellet the cells, and washed with 0.85% NaCl. The supernatants were removed and discarded. Centrifugation and washing procedures were repeated twice. The pellets were re-suspended with 1 mL of 70% isopropyl alcohol and incubated at room temperature for 60 min mixing every 15 min, getting DCS ready for the staining.

### 2.3. Non-Heat-Treated (nTCS) and Heat-Treated (TCS) Cell Suspensions

To begin, 1 mL aliquots from the microorganism’s cultures were dispensed into microcentrifuge tubes and centrifuged at 10,000 *g* for 3 min. The pelleted cells were washed with 0.85% NaCl and the supernatants were removed and discarded. Centrifugation and washing procedures were repeated twice. The pellet was re-suspended with 1 mL of 0.85% NaCl, giving a non-heat-treated cell suspension (nTCS), and an aliquot was used for viable cell count as mentioned above [42].

For the heat treatment viability assays, 10 mL from broth cultures were placed in 50 mL screw-cap glass tubes and heated at 60, 65, or 100 °C for 5 min and immediately introduced in a water bath at 4 °C for 5 min. As *C. sakazakii* mean thermal inactivation D_60 °C_ and D_65 °C_ values are about 4.3 and 0.6 min, respectively (z-value of 5.6 °C; [43]), partial thermal inactivation and sublethal damaged cells resembling under-pasteurization heating conditions were expected after both 60 or 65 °C heat treatments. After the 100 °C treatment, irreversibly destroyed cells were expected, resembling high pasteurization conditions. After the heat treatments, 1 mL of each of the heat-treated cultures were dispensed into microcentrifuge tubes, centrifuged, and washed twice with 0.85% NaCl, as previously described, obtaining heat-treated cell suspensions from each temperature (TCS_60_, TCS_65_, and TCS_100_). The resulting viable cell concentrations were determined as described for the nTCS.

Finally, cell suspensions were mixed into FC analysis tubes at different nTCS:TCS ratios (vol:vol): 100, 75, 50, 25, and 0% of nTCS. Control tubes with only 0.85% NaCl were also prepared.

### 2.4. FC Analysis

Cell suspensions were stained using the Live/Dead BacLight Bacterial Viability and Counting Kit (Molecular Probes, Invitrogen, CO, USA) according to the manufacturer’s instructions as follows: 10 µL of the bacterial cell suspension, 987 µL of 0.85% NaCl, 1.5 µL of 3.34 mM SYTO9 green fluorescent nucleic acid stain, and 1.5 µL of 30 mM propidium iodide (PI) red fluorescent nucleic acid stain were dispensed into each FC tube. For accurate counting, the total volume in the FC tubes was 1000 µL. The final population of the microorganisms should be calculated taking into account the 100-fold dilution into the FC tubes. The tubes were incubated at room temperature for 15 min and were protected from light, as they were wrapped with aluminium foil and stored in a cabinet.

FC analyses were conducted using a Cytomics FC 500 (Beckman Coulter, Brea, CA, USA) with an excitation wavelength of 488 nm from a blue argon laser. Each cell (called event) was characterized by two fluorescent parameters that measured green fluorescence emission (FL1 channel) and red fluorescence emission (FL3 channel). Green fluorescing SYTO9 can enter all cells and is used for assessing total cell counts, whereas red fluorescing PI only enters cells with damaged cytoplasmic membranes. Optical bandpass filters were set up to measure the green fluorescence of SYTO9 at 525/40 nm (FL1; SYTO9 FL1 Log) and the red fluorescence of PI at 620/30 nm (FL3; PI FL3 Log). The measurements were conducted after 300 s or 500,000 events at a low flow rate (10 µL/min).

### 2.5. FC Events versus Plate Counts

Correlations between the FC method and plate counts were calculated [44]. Different cell concentrations of *C. sakazakii* between 10^4^ and 10^9^ CFU/mL were analyzed using the FC and plate count method.

## 3. Results

### 3.1. DCS

**Figure 1:**
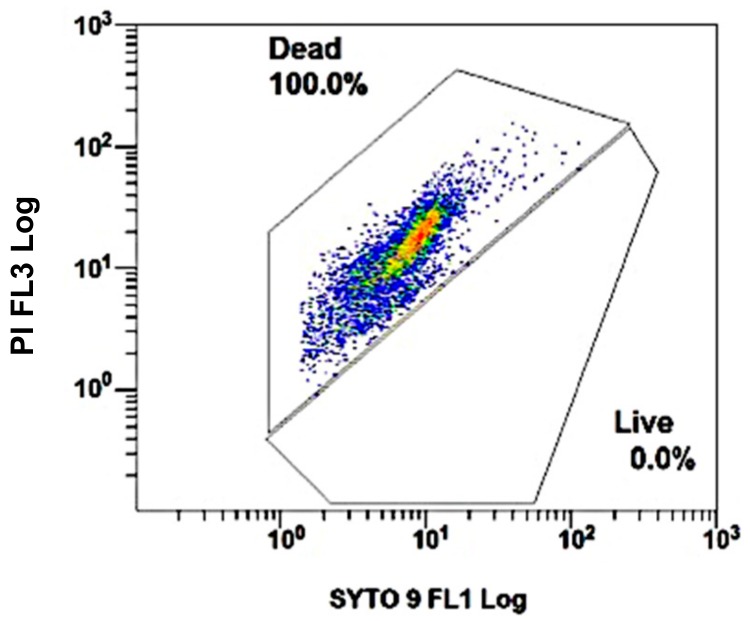
Flow cytometry plot of SYTO9-PI stained alcohol-treated dead cells of Cronobacter sakazakii. The distribution of the observed events was a function of forward and side light scatter Results from one of the assays carried out in triplicate.

The FC plot that was obtained from *C. sakazakii* dead-cells suspensions is shown in Figure 1. The events are distributed according to fluorescence intensity at the two wavelengths that were studied. A gate was positively identified for dead cells since all of them were restricted to a very well-defined area, allowing its use as a control for future differentiation between dead and live cells in different areas.

### 3.2. nTCS and TCS

**Figure 2:**
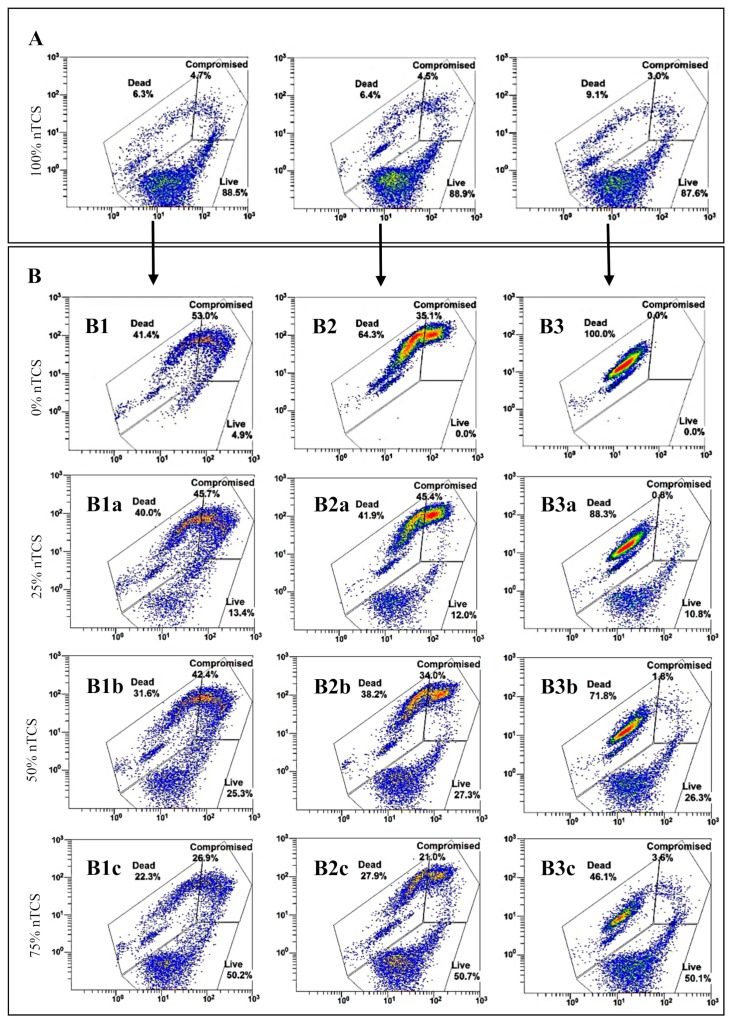
Flow cytometry plots of SYTO9-PI stained *Cronobacter sakazakii* cells from non-treated cell suspensions (**A**: 100% nTCS) and treated cell suspensions (**B1**–**3**: 0% nTCS, i.e., TCS). Different ratios of nTCS:TCS are shown (0, 25, 50, or 75% nTCS) after heat treatments at 60 (**B1a**–**c**), 65 (**B2a**–**c**) or 100 °C (**B3a**–**c**). The plots show gates for live, compromised, and dead cells. Axis X: SYTO9 FL1 Log. Axis Y: PI FL3 Log. Results from one of the assays carried out in triplicate.

Figure 2 shows the distribution of the events that were observed in the cytometer according to the staining of nTCS and different nTCS:TCS ratios. Figure 2A shows three different 100% nTCS plots before they had been heat treated. An area for the live cells was identified. Figure 2B shows the nTCS:TCS mixtures: 0, 25, 50, or 75% of nTCS cultures mixed with TCS previously heat-treated at 60, 65, or 100 °C. After the 100 °C heat treatment (TCS100), dead cells were restricted to a very well-defined area (Figure 2(B3); 0% nTCS or 100% TCS), as previously observed from DCS controls, which are shown in Figure 1. The identification of live cells and dead cells areas allowed the identification of the third area for cells with compromised membranes, which was stained with both colourants. Compromised cells (CC) spread themselves into the intact-membrane (live cells) and damaged-membrane (dead cells) areas, as is observed with the different nTCS:TCS mixtures. When only TCS cells were studied, the number of CC decreased as the heat-treatments’ temperature increased from 60 to 65 °C (Figure 2(B1,B2), respectively) and were not detected at 100 °C (Figure 2(B3)). Furthermore, a small number of live cells were detected in TCS60 (Figure 2 (B1)) but in TCS65 or TCS100 (Figure 2(B2,B3), respectively), showing that the 60 °C treatment does not kill all bacteria, resulting in a high number of CC.

When the percentage of nTCS cells (live cells) was increased in the nTCS:TCS100 mixtures (Figure 2(B3a–c)), the number of CC slightly increased, showing that some of the live cells have compromised membranes—dead cells or TCS100 were detected in a well-defined area without a shift towards the CC’s area (Figure 2(B3)). The number of CC increased with cells coming from both nTCS and TCS65 areas in nTCS:TCS65 mixtures (Figure 2(B2a–c)). This CC’s rise was even more evident in the case of TCS60 in nTCS:TCS6o mixtures (Figure 2(B1a–c)).

### 3.3. FC Events versus Plate Counts

**Figure 3:**
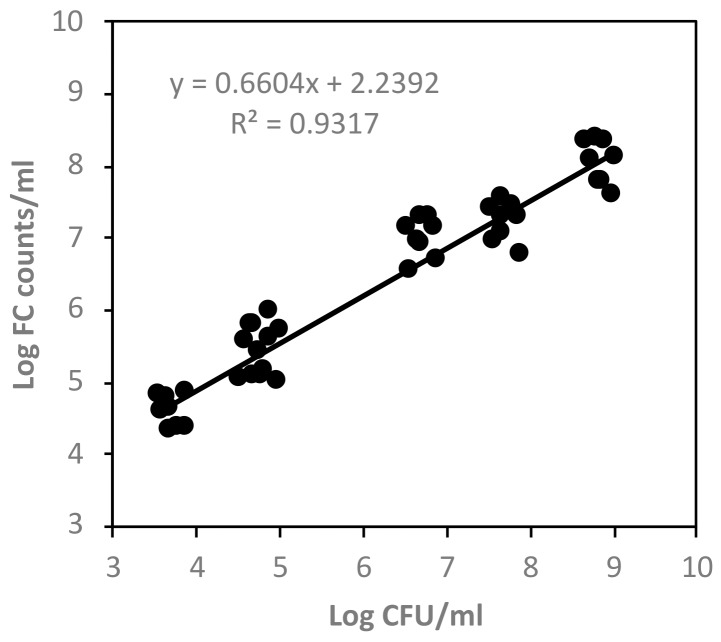
Correlation between the agar plate counts (log CFU/mL) of *C. sakazakii* and total bacterial counts detected by flow cytometry (log FC counts/mL).

Linear regression analysis between FC events of live cells and plate counts was carried out, obtaining a strong correlation (R^2^ = 0.932) (Figure 3).

## 4. Discussion

*C. sakazakii* is a pathogenic bacterium that is able to produce capsular material and biofilms [40,45]. This ability has public health implications because biofilms are usually pathogenic and can cause foodborne outbreaks as well as nosocomial infections [46]. These characteristics allow the microorganism to protect itself from hostile environments—such as food industries and/or hospitals—growing on food matrixes and food industry infrastructures as well as in medical devices [46,47,48]. Three different heat treatments were used to assess the viability of *C. sakazakii* cells, and CC were found. The number of CC slightly increased as the percentage of nTCS (live cells) was gradually increased in the nTCS:TCS_100_ mixtures. The number of live bacteria without compromised membranes in a cell suspension will depend on the environmental conditions affecting their physiological state. Indeed, each cell has its characteristics, behaviour, and response to the environment. Following previous statements [49,50], the kinetics of a bacterial population can be characterized by the extracellular environment and the intracellular conditions. To know how and when cells enter a compromised physiological state will require further investigation and it is far from the aim of the present study. The results were different in nTCS:TCS_60_ and nTCS:TCS_65_ mixtures, i.e., the number of CC increased as a result of cells, with compromised membranes shifting from TCS, i.e., the used temperatures were not completely effective at killing all bacteria but were effective at compromising their membranes and subsequently, their capacity to grow on agar forming colonies and be detected with conventional analyses.

SYTO9 is a hydrophobic cell-permeant nucleic acid stain that shows a large fluorescence enhancement upon binding nucleic acids after penetrating the cell intact membrane [19,27,51]. PI is a membrane impermeant hydrophilic stain with a high molecular weight that binds to nucleic acids only when the cell membrane has pores [27,52,53]. When the PI is mixed with the SYTO9, cells with intact membrane fluoresce green, while cells with damaged membrane fluoresce red—they lose their green fluorescence because the PI competes for the same target areas than the SYTO9, which is a reason behind its greater affinity for nucleic acids than SYTO9 forcing its displacement from its binding [25,27,51,53]. According to Berney et al. [54], microscopy allows one to distinguish the difference between green or red fluorescent cells, however FC allows us to observe a curve-shaped pattern of fluorescence with different amounts of both stains. A third green-red fluorescent group of cells can be detected [23,25,30,55,56], which represents an intermediate state in the permeabilization of the cell membrane, allowing the PI to penetrate the cell but not in enough quantities to efficiently displace the SYTO9 from its binding to the nucleic acids [23,30,57,58,59,60]. Indeed, intermediate states reflecting physiological heterogeneity of cells have been observed [22,23,25,34,54,55,56,57,61,62,63,64,65]. As previously reported [54], the region of intermediate states—with high green and red fluorescence intensity—could have an impact in both the decision making process and the interpretation of results, e.g., the effectiveness of disinfection methods or the counts of viable bacteria in food systems [23,54,55].

SYTO9 has the ability (i) to penetrate both live and dead cells, (ii) it can better penetrate dead cells than live cells because of their damaged membrane, and (iii) it can better penetrate compromised cells than live cells because of their wider membrane pores. PI has the ability (i) to penetrate dead cells because of their damaged membrane, (ii) it can penetrate compromised cells with some limitations due to its high molecular weight and the size of the membrane pores, and (iii) it can compete with SYTO9 for binding the nucleic acids into the dead and/or compromised cells. Due to these facts and the combination of both dyes, the fluorescence intensity of compromised cells is slightly higher than the dead cells intensity (axis Y, Figure 2B1 and/or Figure 2B2) and it is slightly higher than the live cells intensity (axis X, Figure 2B1 and/or Figure 2B2). Moreover, *C. sakazakii* cells linked high quantities of both dyes, showing higher signals than the live and/or dead cells because of the high amount of nucleic acids in the interior of the cells and in the exterior around them (compromised cells have problems in the structure of their membranes and high amounts of extracellular nucleic acids can be detected after its staining, contributing to the fluorescence intensity).

Berney et al. [54] related the mentioned intermediate area with different concentrations of dyes in the interior of the cells, attributing the lead role to the intracellular SYTO9 concentration—the intracellular PI concentration had a low impact on the increase in green and red fluorescence intensity according to these authors.

Recently, Rosenberg et al. [66] reported similar subpopulations of double-stained (SYTO9 plus PI) *Escherichia coli* cells from a biofilm. These authors were able to detect cells with green interiors and red surfaces; the latter probably due to the staining of extracellular nucleic acids by PI. These authors have identified this subpopulation as a false dead population with increased green and red fluorescence intensity.

To the best of our knowledge, the nature of this intermediate state has not been clarified in *C. sakazakii*, probably due to the technical sophistication of the techniques and the associated costs. In the present study, we have detected such a state and linked it to the physiological properties of the bacterial cells. We have observed a distinctive curve-shaped pattern of fluorescence for *C. sakazakii* cells suspensions that are exposed to heat treatments. The cloud of live cells followed a curve-shaped pattern, with events shifting through the dead cells area, reflecting the different physiological states of Gram-negative bacteria as the damage of the cells proceeds. A first step would be the damage of the outer membrane while the cytoplasmic membrane is still intact, as PI cannot penetrate it, followed by a second step with the complete damage of the cytoplasmic membrane. Berney et al. [54] reported a similar pattern when Gram-negative bacterial cells such as *Escherichia coli, Salmonella enterica* serovar Typhimurium, and *Shigella flexneri* were UVA-irradiated or EDTA-treated, indicating that the outer membrane of late stationary-phase cells of Gram-negative bacteria is a barrier for SYTO9. The permeabilization of the outer membrane can be done with artificial UV [54,63,67,68] as well as sunlight [63] before the disruption of the cytoplasmic membrane. Our results would show the disruption of the outer membrane by mild heat treatments followed by some grade of permeabilization of the cytoplasmic membrane. This phenomenon was not observed in Gram-positive *Enterococcus faecalis* [54], which lacks an outer membrane.

*C. sakazakii* may be present in reconstituted PIF and may possibly survive the mild heat stress associated with its reconstitution [41] because clinical strains appeared to be more thermotolerant than their environmental counterparts [69]. Furthermore, cross-contamination can occur, resulting in the presence of cells that were not subjected to the heat treatment that is used for PIF reconstitution [41]. Recommendations for the preparation of PIF in the neonatal intensive care units or for bottle-feeding at home were proposed by the FAO/WHO [14] to ensure safety in PIF: boiled water has to be cooled down up to 70 °C for 30 min. Despite these recommendations, the instructions for reconstitution may suggest using water at temperatures as low as 40 °C [41,70,71] due to undesirable effects on the organoleptic and nutritional properties of reconstituted PIF [72] or to avoid the risk of severe burns [73]. Furthermore, a moderate reheating (35–40 °C) of previously reconstituted PIF or a mild heat treatment (40–70 °C) of the water intended for the reconstitution of PIF should not be discarded, especially at home, as these situations would represent high-risk food safety scenarios for children [73] because untreated and/or mildly heat-treated cells may recover and grow during the holding time [71]. In the present study, three heat treatments were used. The 100 °C heat-treated samples did not show bacterial growth on agar plates nor live cells in the flow cytometer; however, treatments at 60 or 65 °C did not eliminate all bacteria, giving counts of about 4 to 5 log CFU/mL in agar plates and 5.1–5.6 log events/mL in the flow cytometer. These results show that mild heat treatments are not high enough to guarantee the safety of PIF and stress the importance of proper boiling of water prior to its use for reconstituting PIF. Furthermore, a cooling period under sterilized conditions until an adequate temperature for consumption is achieved should be carried out. Parra-Flores et al. [41] arrived at a similar conclusion after studying the variability in cell response of *C. sakazakii* after mild heat treatments at 50 °C for 5 or 10 min.

Several authors [15,17,19,20] stated that the membrane integrity reflecting the viability of the cell appears to be dependent on both environmental conditions and the physiological status of the cell during the time of analysis. Among the environmental conditions, temperature, time, and different compounds have a great influence on the viability of the cells: Arku et al. [15] showed that *C. sakazakii* cell survival decreases as the temperature increases from 52 to 67 °C for 30 min, Choi et al. [38] found that the proportion of *C. sakazakii* cells in the PI fluorescent region increases under the influence of antimicrobial compounds as a function of the time for 5, 10, or 30 min, and Marty et al. [74] reported that a large fraction of the proteome of *Halobacterium salinarum* was strongly disrupted under heat stress from 40 to 60 °C for 60 min. Similar results were found for *Escherichia coli* [75]. The ability of a *C. sakazakii* toxin to be able to remain stable at 90 °C for 30 min needs to be addressed [5].

The physiological status of the cells is very important for the integrity of its membrane. Parra-Flores et al. [41] observed high variability in the lag phase of *C. sakazakii* populations due to both a previous heat-shock of the cells and the subsequent growth temperature; these results agree with previous findings regarding *Listeria innocua, Enterococcus faecalis, Salmonella enterica*, and *Pseudomonas fluorescens* [70,76,77,78,79,80].

FC analyses cell by cell quickly and precisely give quantitative information about the heterogeneity of the physiological state of the population [22,25,30,55,56,81]. Compromised cells cannot be detected using classical plate count methods [33,34,35] and unfortunately, from a public health point of view, they can retain their pathogenic potential. The ability of pathogenic bacteria to recover after different treatments has been reported: *Escherichia coli* O157:H7 were recovered during the refrigerated storage of ready-to-eat pasta salads [30], *Bacillus cereus* and *B. weihenstephanensis* after assessing their acid shock viability [22], *Legionella pneumophila* and *Escherichia coli* were recovered after heat treatments [82], and *Staphylococcus epidermidis* and *E. coli* were retrieved from biofilms [66].

## 5. Conclusions

Despite the recommendations, several situations could arise such as the reconstitution of PIF with water at 40–70 °C or even the use of a mild re-heating treatment of a previously reconstituted PIF. All these situations dramatically increase the risk associated with the presence of *C. sakazakii* in reconstituted PIF. Mild heat treatments at 60 or 65 °C did not eliminate all bacteria, so the proper use of boiled water for reconstituting PIF must be emphasized. Furthermore, high numbers of compromised cells were detected after the mild heat treatments; flow cytometry was able to differentiate the compromised cells, which were viable but non-culturable, from live and/or dead cells. Classic culture techniques are time-consuming and do not show the physiological state of the cells, so samples with compromised cells—maintaining their pathogenic characteristics—could be consumed by children. A screening test of the bottles of formulae with flow cytometry prior to administration to the neonates in the neonatal intensive care units could help to discriminate those samples showing compromised cells, which could represent a risk to the infant.
